# Contagious Affections

**Published:** 1888-08

**Authors:** 


### CONTAGIOUS AFFECTIONS.

Should the present rate of increase in the list of contagious affections be maintained for any great length of time, who can feel secure? The number of those that have for years been known to be contagious, to say nothing of the infections, has been just cause for apprehension, but when there have been added to those the numbers that modern bacteriologists are constantly pointing out to us, and the many avenues by which they enter our bodies, the array seems appalling. Not only does man convey them to his fellowman, but the inferior animals upon which we are so much dependent, and by which we are constantly sourrounded, seem to be so many storehouses filled with pent-up danger to man. The cow, that gives the milk to nourish the child when the mother's fails, must, it seems, transfer to the infant the bacilli of the tuberculosis. The cat with which that child would amuse itself must convey to it the poison of diphtheria. The dog that is the boy's faithful companion not only causes hydrophobia but other dangers through the minute bacteria which he conveys to his confiding playmate. Even man's most trusted horse may give him glanders or actinomycosis as well as other affections; and the animals on which man largely subsists must convey to him trichinæ or other dreadful, even if invisible, danger.

Nature seems unable longer to give pure water to drink, but must instil into it sufficient bacilli of typhoid from unknown or unsuspected sources to cause numerous deaths.

Since freezing the water into ice will not destroy those bacilli, nor freezing cream into the refreshing ice-cream give assurance but that those of tuberculosis have been awaiting that opportunity to enter our bodies by the side of Vaughan's tyrotoxicon, whither shall man betake himself that some of these microscopic fiends will not be found in his wake, assuring him, as of old, that where thou goest I will go and where thou lodgest I will lodge.

If our modern investigators continue much longer their revelations no man can feel safe until he has secured for himself a lodging place in a modern crematory.

